# Supplementary material for: The Role of Insulin-like Peptide in Maintaining Hemolymph Glucose Homeostasis in the Pacific White Shrimp Litopenaeus vannamei
Source: Int J Mol Sci. 2022 Mar 17;23(6):3268. doi: 10.3390/ijms23063268 (PMC8948857; doi:10.3390/ijms23063268)
Supplement: Supplementary file 1 [file ijms-23-03268-s001.zip › Supplementary SA. File SA1. Author comments concerning the Ovarian data previously published.pdf]

Author comments concerning the Ovarian data previously published.

The authors have reprocessed the data previously published under the following reference:

Hocker JR, Bishop EA, Lightfoot SA, Lerner MR, Peyton MD, Brackett DJ, Hanas RJ, McMeekin DS, Walker JL, Hanas JS. Serum profiling to distinguish early- and late-stage ovarian cancer patients from disease-free individuals. Cancer Invest. 2012 Feb;30(2):189-97. doi: 10.3109/07357907.2011.636115. Epub 2011 Dec 7. PubMed PMID: 22149058.

The data utilized for the indicated publication have been normalized under different parameters than previously published. The current data processing, normalization and peak identification, is identical for all samples within this work.
