# Supplementary material for: The Role of Insulin-like Peptide in Maintaining Hemolymph Glucose Homeostasis in the Pacific White Shrimp Litopenaeus vannamei
Source: Int J Mol Sci. 2022 Mar 17;23(6):3268. doi: 10.3390/ijms23063268 (PMC8948857; doi:10.3390/ijms23063268)
Supplement: Supplementary file 1 [file ijms-23-03268-s001.zip › Supplementary SA. File SA2. Patient samples utilized for MSMS.pdf]

Patient samples utilized for MS/MS		
test	pathology	deidentified PATIENT ID
12V34	Endometrial Cancer I & II	37
12V34	Endometrial Cancer I & II	42
12V34	Endometrial Cancer I & II	46
12V34	Endometrial Cancer I & II	48
12V34	Endometrial Cancer I & II	51
12V34	Endometrial Cancer III & IV	60
12V34	Endometrial Cancer III & IV	63
12V34	Endometrial Cancer III & IV	67
12V34	Endometrial Cancer III & IV	68
12V34	Endometrial Cancer III & IV	70
12VBN	Endometrial Cancer I & II	36
12VBN	Endometrial Cancer I & II	39
12VBN	Endometrial Cancer I & II	40
12VBN	Endometrial Cancer I & II	44
12VBN	Endometrial Cancer I & II	45
12VBN	Benign gynecology	81
12VBN	Benign gynecology	84
12VBN	Benign gynecology	88
12VBN	Benign gynecology	90
12VBN	Benign gynecology	93
34VBN	Endometrial Cancer III & IV	60
34VBN	Endometrial Cancer III & IV	66
34VBN	Endometrial Cancer III & IV	67
34VBN	Endometrial Cancer III & IV	68
34VBN	Endometrial Cancer III & IV	72
34VBN	Benign gynecology	75
34VBN	Benign gynecology	77
34VBN	Benign gynecology	80
34VBN	Benign gynecology	84
34VBN	Benign gynecology	87
