# Supplementary material for: The Role of Insulin-like Peptide in Maintaining Hemolymph Glucose Homeostasis in the Pacific White Shrimp Litopenaeus vannamei
Source: Int J Mol Sci. 2022 Mar 17;23(6):3268. doi: 10.3390/ijms23063268 (PMC8948857; doi:10.3390/ijms23063268)
Supplement: Supplementary file 1 [file ijms-23-03268-s001.zip › Supplementary SA. File SA3. Binary LOOCV test metrics.pdf]

Table : Patient Characteristics by Group Analyzed

Figure	Test Metrics (group 1 vs. group 2)	% LOOCV Mean (SD) group 1	% LOOCV Mean (SD) group 2	True Positive group 1	False Positive group 2	True Negative group 2	False Negative group 1	N
2	Endometrial Cancer Stages I, II, III & IV vs. Abnormal Endometrium Non-Cancer; ROC(area=0.95)	50.0 (6.0)	28.0 (4.8)	39/40 (98%)	0/20 (0%)	20/20 (100%)	1/40 (2%)	Endometrial Cancer Stages I, II, III & IV, N=40 Abnormal Endometrium Non-Cancer, N=20
3	Endometrial Cancer Stages I & II vs. Abnormal Endometrium Non-Cancer; ROC(area=1.0)	58.5 (5.3)	34.3 (4.9)	20/20 (100%)	0/20 (0%)	20/20 (100%)	0/20 (0%)	Endometrial Cancer Stages I & II, N=20 Abnormal Endometrium Non-Cancer, N=20
4	Endometrial Cancer Stages III & IV vs. Abnormal Endometrial Not-Cancer; ROC(area=1.0)	59.9 (4.5)	41.0 (4.2)	20/20 (100%)	0/20 (0%)	20/20 (100%)	0/20 (0%)	Endometrial Cancer Stages III & IV, N=20 Abnormal Endometrial Not-Cancer, N=20
5	Endometrial Cancer Stages I & II vs. Endometrial Cancer Stages III & IV; ROC(area=1.0)	60.1 (5.9)	36.7 (5.6)	20/20 (100%)	0/20 (0%)	20/20 (100%)	0/20 (0%)	Endometrial Cancer Stages I & II, N=20 Endometrial Cancer Stages III & IV, N=20
6	Ovarian Cancer Stages I, II, III & IV vs. Endometrial Cancer Stages I, II, III & IV; ROC(area=1.0)	69.9 (6.5)	42.0 (4.3)	0/35 (100%)	0/20 (0%)	35/35 (100$)	0/20 (0%)	Ovarian Cancer Stages: I, N=16; II, N=4; III,N=12; IV,N=2 Endometrial Cancer Stages I, N=19; II, N=1; III, N=19; IV, N=1

Table : Significance Between Tested Groups

Cohen's *d*	Figure	Test Metrics	Sensitivity	Efficiency/ {accuracy}	True Positive Rate	False Positive Rate	Specificity	*P*-value	Random database *P*-value	ROC (Area)	Selection Frequency Minimum	# peaks	m/Z range
4.0	2	Endometrial Cancer Stages I, II, III & IV vs. Abnormal Endometrium Non-Cancer	0.98	0.98	0.98	0	1.0	6.6 x 10 -20	0.014	0.95	0.50	96-104	400-1800
4.7	3	Endometrial Cancer Stages I, II, III & IV vs. Abnormal Endometrium Non-Cancer	1.0	1.0	1.0	0.0	1.0	8.8 x 10 -18	0.03	1.0	0.75	111-1119	400-2000
3.9	4	Endometrial Cancer Stages III & IV vs. Abnormal Endometrial Not-Cancer	1.0	1.0	1.0	0.0	1.0	1.6 x 10 -16	0.05	1.0	0.40	113-124	500-2000
4.0	5	Endometrial Cancer Stages I & II vs. Endometrial Cancer Stages III & IV	1.0	1.0	1.0	0.0	1.0	8.9 x 10 -16	0.026	1.0	0.50	93-102	450-1800
5.0	6	Ovarian Cancer Stages I, II, III & IV vs. Endometrial Cancer Stages I, II, III & IV	1.0	1.0	1.0	0.0	1.0	6.8 x 10-29	0.21	1.0	0.98	351	400-2000

Table: Patient Characteristics

Groups	Age (Range) SD	BMI (Range) SD	Tobacco History	ETOH History	Diabetes	HTN	CAD	Arthritis	NSAIDs	ASA	Metformin	Insulin
Abnormal Endometrium (control), N=20	57.0 (50 - 64) 5.1	35.3 (22 - 54) 8.1	4	6	1	10	1	1	3	2	0	0
Endometrial Cancer, N=40	57.9 (31 - 77) 8.4	35.2 (21 - 48) 6.7	13	6	6	21	1	7	14	4	2	1
Stage I, N=19	55.8 (51 - 61) 2.6	36.6 (24 - 48) 6.0	6	3	3	11	1	1	5	4	1	0
Stage II, N=1	57.0 (57 - 57) na	21.0 (21 -20) na	1	0	0	1	0	1	1	0	0	0
Stage III, N=19	61.1 (31 - 77) 10.5	34.2 (22 - 45) 6.5	6	3	3	9	0	4	8	3	1	1
Stage IV, N=1	40.0 (40 -40) na	42.0 (42 - 42) na	0	0	0	0	0	1	0	0	0	0
Ovarian Cancer, N=34	60.6 (35 - 78) 9.9	29.0 (18 - 53) 9.2	8	7	4	16	3	3	8	5	1	0
Stage I, N=16	58.8 (45 - 73) 7.5	31.7 (18 - 53) 10.3	6	6	3	8	2	3	6	2	1	0
Stage II, N=4	72.3 (67 - 76) 3.8	24.0 (19 - 27) 3.8	2	0	0	2	0	0	0	0	0	0
Stage III, N=12	59.7 (35 - 78) 11.3	26.0 (18 - 43) 7.0	0	1	0	4	0	0	2	2	0	0
Stage IV, N=2	57.5 (45 - 70) 17.7	35.5 (25 - 46) 14.8	0	0	1	2	1	0	0	1	0	0
