# Supplementary material for: The Role of Insulin-like Peptide in Maintaining Hemolymph Glucose Homeostasis in the Pacific White Shrimp Litopenaeus vannamei
Source: Int J Mol Sci. 2022 Mar 17;23(6):3268. doi: 10.3390/ijms23063268 (PMC8948857; doi:10.3390/ijms23063268)
Supplement: Supplementary file 1 [file ijms-23-03268-s001.zip › Supplementary SA. File SA4. Concerning Files uploaded to JPost repository.pdf]

Concerning Files uploaded to JPost repository

Excel files are in a 64 bit format and are not readable using the 32bit Excel program.

The “working calculations LOOCV example file.xlsx” is fully functional with active equations (be careful if you change data or try to edit the file). This file is annotated on the “answers” and the “LOOCV_1_1” worksheets to detail how it works. Graphs contained in this file will change as you change parameters.
