# Supplementary material for: The Role of Insulin-like Peptide in Maintaining Hemolymph Glucose Homeostasis in the Pacific White Shrimp Litopenaeus vannamei
Source: Int J Mol Sci. 2022 Mar 17;23(6):3268. doi: 10.3390/ijms23063268 (PMC8948857; doi:10.3390/ijms23063268)
Supplement: Supplementary file 1 [file ijms-23-03268-s001.zip › Supplementary SA. File SA5. LCQ-ADVANTAGE Instrument Method for MSMS example.pdf]

LCQ-ADVANTAGE Instrument Method
Creator: Administrator
Last modified: 10/5/2015 by Administrator
MS Run Time (min): 100.00
Sequence override of method parameters not enabled.
Divert Valve: not used during run
Contact Closure: not used during run
MS Detector Settings:
Segment 1 Information
Duration (min): 5.00
Number of Scan Events: 1
Tune Method: msms_sera_settings_
Scan Event Details:
1: + c o(150.0-2000.0)
Segment 2 Information
Duration (min): 5.00
Number of Scan Events: 1
Tune Method: Sera_MSMS
Scan Event Details:
1: + c ·(506.0)->o(135.0-2000.0)
MS/MS: CE 35.0% IsoW 1.5
Segment 3 Information
Duration (min): 5.00
Number of Scan Events: 1
Tune Method: Sera_MSMS
Scan Event Details:
1: + c ·(521.0)->o(140.0-2000.0)
MS/MS: CE 35.0% IsoW 1.5

Segment 4 Information
Duration (min): 5.00
Number of Scan Events: 1
Tune Method: Sera_MSMS
Scan Event Details:
1: + c ·(525.0)->o(140.0-2000.0)
MS/MS: CE 35.0% IsoW 1.5
Segment 5 Information
Duration (min): 5.00
Number of Scan Events: 1
Tune Method: Sera_MSMS
Scan Event Details:
1: + c ·(528.0)->o(145.0-2000.0)
MS/MS: CE 35.0% IsoW 1.5
Segment 6 Information
Duration (min): 5.00
Number of Scan Events: 1
Tune Method: Sera_MSMS
Scan Event Details:
1: + c ·(541.0)->o(145.0-2000.0)
MS/MS: CE 35.0% IsoW 1.5
Segment 7 Information
Duration (min): 5.00
Number of Scan Events: 1
Tune Method: Sera_MSMS
Scan Event Details:
1: + c ·(549.0)->o(150.0-2000.0)
MS/MS: CE 35.0% IsoW 1.5
Segment 8 Information
Duration (min): 5.00
Number of Scan Events: 1
Tune Method: Sera_MSMS
Scan Event Details:
1: + c ·(558.0)->o(150.0-2000.0)
MS/MS: CE 35.0% IsoW 1.5
Segment 9 Information
Duration (min): 5.00
Number of Scan Events: 1
Tune Method: Sera_MSMS
Scan Event Details:
1: + c ·(559.0)->o(150.0-2000.0)
MS/MS: CE 35.0% IsoW 1.5
Segment 10 Information
Duration (min): 5.00
Number of Scan Events: 1
Tune Method: Sera_MSMS
Scan Event Details:
1: + c ·(584.0)->o(160.0-2000.0)
MS/MS: CE 35.0% IsoW 1.5
Segment 11 Information
Duration (min): 5.00
Number of Scan Events: 1
Tune Method: Sera_MSMS
Scan Event Details:
1: + c ·(603.0)->o(165.0-2000.0)
MS/MS: CE 35.0% IsoW 1.5
Segment 12 Information
Duration (min): 5.00
Number of Scan Events: 1
Tune Method: Sera_MSMS
Scan Event Details:
1: + c ·(618.0)->o(170.0-2000.0)
MS/MS: CE 35.0% IsoW 1.5
Segment 13 Information
Duration (min): 5.00
Number of Scan Events: 1

Tune Method: Sera_MSMS
Scan Event Details:
1: + c ·(638.0)->o(175.0-2000.0)
MS/MS: CE 35.0% IsoW 1.5
Segment 14 Information
Duration (min): 5.00
Number of Scan Events: 1
Tune Method: Sera_MSMS
Scan Event Details:
1: + c ·(649.0)->o(175.0-2000.0)
MS/MS: CE 35.0% IsoW 1.5
Segment 15 Information
Duration (min): 5.00
Number of Scan Events: 1
Tune Method: Sera_MSMS
Scan Event Details:
1: + c ·(665.0)->o(180.0-2000.0)
MS/MS: CE 35.0% IsoW 1.5
Segment 16 Information
Duration (min): 5.00
Number of Scan Events: 1
Tune Method: Sera_MSMS
Scan Event Details:
1: + c ·(683.0)->o(185.0-2000.0)
MS/MS: CE 35.0% IsoW 1.5
Segment 17 Information
Duration (min): 5.00
Number of Scan Events: 1
Tune Method: Sera_MSMS
Scan Event Details:
1: + c ·(733.0)->o(200.0-2000.0)
MS/MS: CE 35.0% IsoW 1.5
Segment 18 Information
Duration (min): 5.00
Number of Scan Events: 1
Tune Method: Sera_MSMS
Scan Event Details:
1: + c ·(761.0)->o(205.0-2000.0)
MS/MS: CE 35.0% IsoW 1.5
Segment 19 Information
Duration (min): 5.00
Number of Scan Events: 1
Tune Method: Sera_MSMS
Scan Event Details:
1: + c ·(763.0)->o(210.0-2000.0)
MS/MS: CE 35.0% IsoW 1.5
Segment 20 Information
Duration (min): 5.00
Number of Scan Events: 1
Tune Method: Sera_MSMS
Scan Event Details:
1: + c o(150.0-2000.0)
