# Supplementary material for: The Role of Insulin-like Peptide in Maintaining Hemolymph Glucose Homeostasis in the Pacific White Shrimp Litopenaeus vannamei
Source: Int J Mol Sci. 2022 Mar 17;23(6):3268. doi: 10.3390/ijms23063268 (PMC8948857; doi:10.3390/ijms23063268)
Supplement: Supplementary file 1 [file ijms-23-03268-s001.zip › Supplementary SA. File SA6. LCQ-ADVANTAGE Machine Status Log MSMS example.pdf]

LCQ-ADVANTAGE Machine Status Log	
API SOURCE	
Source Voltage (kV):	1.71
Source Current (uA):	0.19
Source Thermocouple OK:	No
Source Temp (C):	0
Sheath Gas Flow Rate ():	-0.15
Aux/Sweep Gas Flow Rate():	-0.16
Capillary RTD OK:	Yes
Capillary Voltage (V):	-10.4
Capillary Temp (C):	195.2
Tube Lens Voltage (V, set point):	-105
VACUUM	
Vacuum OK:	Yes
Ion Gauge Pressure OK:	Yes
Ion Gauge Status:	On
Ion Gauge (x10e-5 Torr):	1.15
Convectron Pressure OK:	Yes
Convectron Gauge (Torr):	0.86
TURBO PUMP	
Status:	Running
Life (hours):	86746
Speed (rpm):	60000
Power (Watts):	37
ION OPTICS	
Multipole 1 Offset (V):	-3.95
Lens Voltage (V):	-19.62
Multipole 2 Offset (V):	-5.27
Multipole RF Amplitude (Vp-p, set point):	460
Coarse Trap DC Offset (V):	-9.52
Fine Trap DC Offset (V):	-9.63
MAIN RF	
Reference Sine Wave OK:	Yes
Standing Wave Ratio OK:	Yes
Main RF DAC (steps):	63
Main RF Detected (V):	-0.08
RF Detector Temp (C):	32.91
Main RF Modulation (V):	0
Main RF Amplifier (Vp-p):	8.74
RF Generator Temp (C):	35.69

LCQ-ADVANTAGE Machine Status Log	
ION DETECTION SYSTEM	
Dynode Voltage (kV):	-14.85
Multiplier Actual (V):	-1217.25
POWER SUPPLIES	
+5V Supply Voltage (V):	5.05
-15V Supply Voltage (V):	-14.99
+15V Supply Voltage (V):	14.74
+24V Supply Voltage (V):	23.63
-28V Supply Voltage (V):	-27.95
+28V Supply Voltage (V):	28.45
+28V Supply Current (Amps):	1.14
+35V Supply Voltage (V):	36.04
+36V Supply Voltage (V):	35.97
-150V Supply Voltage (V):	-157.11
+150V Supply Voltage (V):	156.23
-205V Supply Voltage (V):	-212.49
+205V Supply Voltage (V):	210.51
Ambient Temp (C):	22.17
INSTRUMENT STATUS	
Instrument:	On
Analysis:	Acquiring
SYRINGE PUMP	
Status:	Ready
DIGITAL INPUTS	
READY IN is active:	No
START IN is active:	No
Divert/Inject valve:	Inject
