# Supplementary material for: The Role of Insulin-like Peptide in Maintaining Hemolymph Glucose Homeostasis in the Pacific White Shrimp Litopenaeus vannamei
Source: Int J Mol Sci. 2022 Mar 17;23(6):3268. doi: 10.3390/ijms23063268 (PMC8948857; doi:10.3390/ijms23063268)
Supplement: Supplementary file 1 [file ijms-23-03268-s001.zip › Supplementary.pdf]

Supplementary SA: Supplementary Information Word Files. File SA1: Author comments concerning the Ovarian data previously published; **File SA2**: Patient samples utilized for MSMS; **File SA3**: Binary LOOCV test metrics; **File SA4**: Concerning Files uploaded to JPost repository; **File SA5**: LCQ-ADVANTAGE Instrument Method for MSMS example; **File SA6**: LCQ-ADVANTAGE Machine Status Log MSMS example).

Supplementary SB: Supplementary Information Excel Files. File SB1: Sera as used in LOOCV Figures; **File SB2**: Master peak areas for each patient De-identified; **File SB3**: LOOCV Significant peaks by test and figure; **File SB4**: LOOCV results of sera MS peaks; **File SB5**: Peak list for MSMS analysis of sera).

Figure S1. LOOCV Analysis of Randomized Groups.
Figure S2. LOOCV Analysis of Stages I or III Ovarian and Endometrial Cancers.
Table S1. Gene IDs and Names of proteins.
Supplementary SA: Supplementary Information Word Files. File SA1: Author comments concerning the Ovarian data previously published; File SA2: Patient samples utilized for MSMS; File SA3: Binary LOOCV test metrics; File SA4: Concerning Files uploaded to JPost repository; File SA5: LCQ-ADVANTAGE Instrument Method for MSMS example; File SA6: LCQ-ADVANTAGE Machine Status Log MSMS example).

Supplementary SB: Supplementary Information Excel Files. File SB1: Sera as used in LOOCV Figures; File SB2: Master peak areas for each patient De-identified; File SB3: LOOCV Significant peaks by test and figure; File SB4: LOOCV results of sera MS peaks; File SB5: Peak list for MSMS analysis of sera).
