# Supplementary figures and images for: The Role of Insulin-like Peptide in Maintaining Hemolymph Glucose Homeostasis in the Pacific White Shrimp Litopenaeus vannamei
Source: Int J Mol Sci. 2022 Mar 17;23(6):3268. doi: 10.3390/ijms23063268 (PMC8948857; doi:10.3390/ijms23063268)

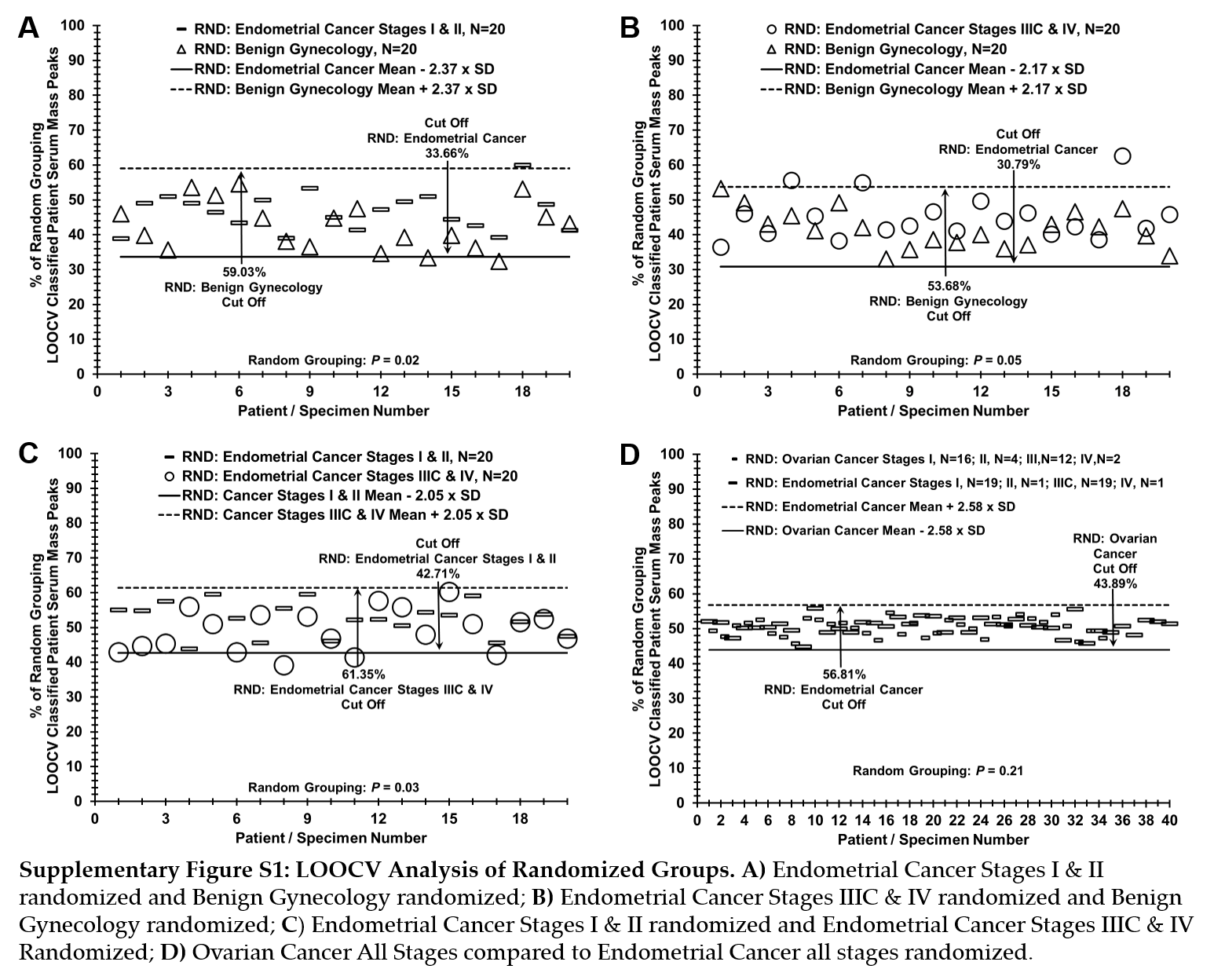

Supplement: Supplementary file 1 [file ijms-23-03268-s001.zip › Supplemental Figure S1.tif]

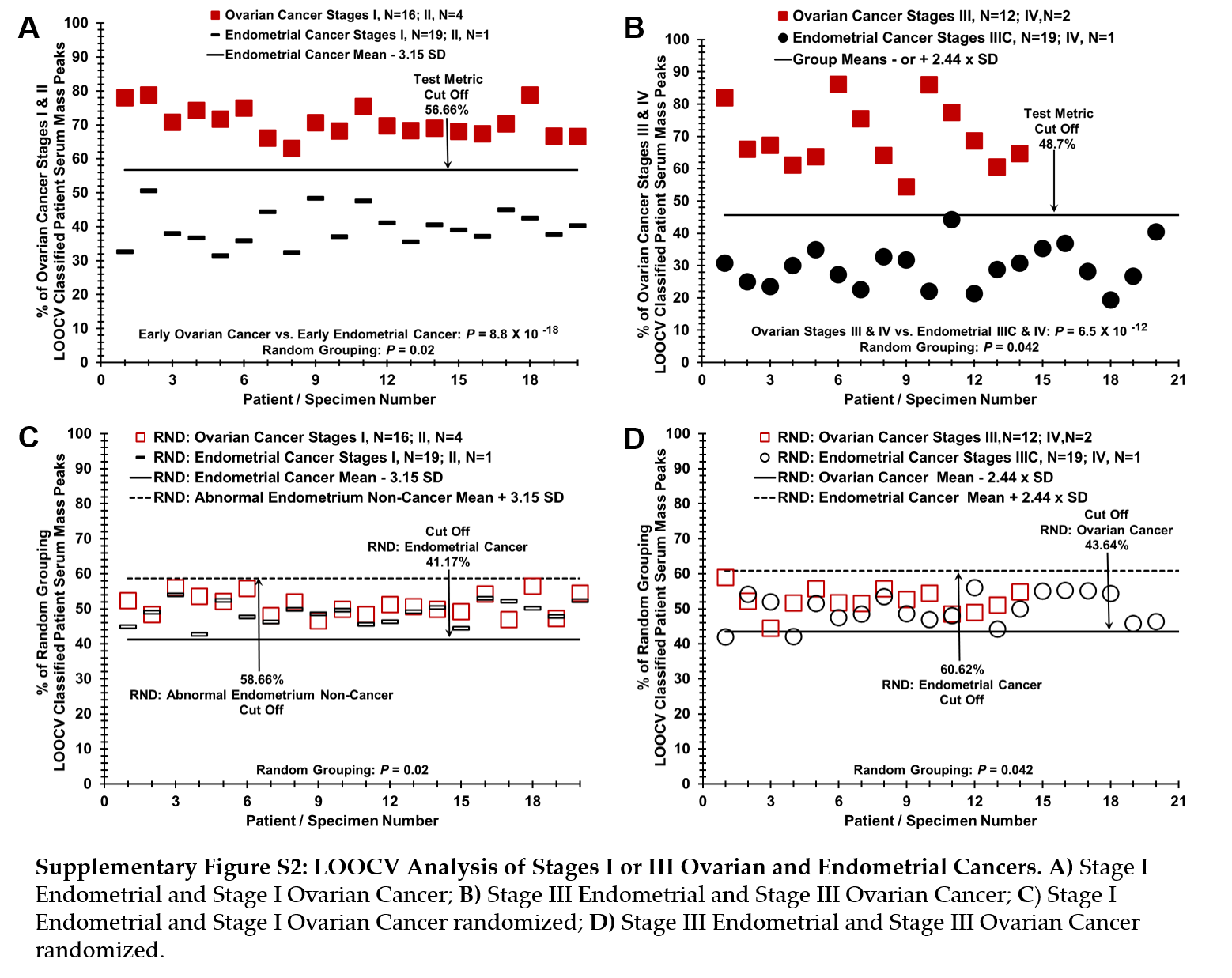

Supplement: Supplementary file 1 [file ijms-23-03268-s001.zip › Supplemental Figure S2.tif]
